# TI-Stan: Model Comparison Using Thermodynamic Integration and HMC

**DOI:** 10.3390/e21121161

**Published:** 2019-11-27

**Authors:** R. Wesley Henderson, Paul M. Goggans

**Affiliations:** Department of Electrical Engineering, University of Mississippi, University, MS 38677, USA; goggans@olemiss.edu

**Keywords:** model comparison, MCMC, thermodynamic Integration, HMC

## Abstract

We present a novel implementation of the adaptively annealed thermodynamic integration technique using Hamiltonian Monte Carlo (HMC). Thermodynamic integration with importance sampling and adaptive annealing is an especially useful method for estimating model evidence for problems that use physics-based mathematical models. Because it is based on importance sampling, this method requires an efficient way to refresh the ensemble of samples. Existing successful implementations use binary slice sampling on the Hilbert curve to accomplish this task. This implementation works well if the model has few parameters or if it can be broken into separate parts with identical parameter priors that can be refreshed separately. However, for models that are not separable and have many parameters, a different method for refreshing the samples is needed. HMC, in the form of the MC-Stan package, is effective for jointly refreshing the ensemble under a high-dimensional model. MC-Stan uses automatic differentiation to compute the gradients of the likelihood that HMC requires in about the same amount of time as it computes the likelihood function itself, easing the programming burden compared to implementations of HMC that require explicitly specified gradient functions. We present a description of the overall TI-Stan procedure and results for representative example problems.

## 1. Introduction

TI is a numerical technique for evaluating model evidence integrals. The technique was originally developed [1] to estimate the free energy of a fluid. Various improvements and changes have been made over the decades, and the incarnation of the technique that our method is based on is the adaptively-annealed, importance sampling-based method described by Goggans and Chi [2]. Their implementation follows John Skilling’s BayeSys [3], and both make use of BSS and the Hilbert curve to complete the implementation. This article proposes a modification of this method that uses PyStan [4,5] and the NUTS [6] instead of BSS and the Hilbert curve. A Python 3 implementation of this method by the authors can be found on GitHub (https://github.com/rwhender/ti-stan) [7]. This article is an adaptation of portions of the first author’s doctoral dissertation [8] (Chapter 3). This article is an expanded version of an article in the proceedings of the 39th International Workshop on Bayesian Inference and Maximum Entropy Methods in Science and Engineering [7].

### 1.1. Motivation

The family of adaptively-annealed TI methods are important for solving model comparison problems in engineering, where we frequently need to evaluate complex physics-based mathematical models. TI methods with fixed annealing schedules (e.g., [9,10]) are useful for solving more traditional statistics problems but tend to fail with the complex models that arise in engineering problems. TI methods that use BSS on the Hilbert curve are useful for a large set of problems; however, these methods see diminishing returns when the number of model parameters grows somewhat large (>10 or so). These performance issues can be mitigated if the model equation can be decomposed into additive components with identical form and equivalent joint priors on their parameters. However, for problems with many model parameters and with model equations that cannot be decomposed, a different class of methods is required.

Nested sampling [11,12] is another widely-used method for evaluating model evidence. One of the advantages of nested sampling is that its estimate of the evidence is not spoiled by an area of convexity in the likelihood function, whereas thermodynamic integration methods can be confounded by such likelihood functions. Skilling restates this difference in his recent conference paper [13], stating without providing examples that this difference constitutes a reason to prefer nested sampling over thermodynamic integration-based methods (also known as simulated annealing-based methods). In our experience, we have not encountered a model that yields a likelihood function with sufficient convexity to confound the thermodynamic integration estimate of the evidence. We have found instead that the biggest problem in implementing model evidence estimation methods lies in devising a method to generate new samples within an equi-likelihood contour that are sufficiently independent for nested sampling to proceed. It is worth noting that the Galilean sampling method advanced in [13] may solve these issues, shifting the balance toward nested sampling again. Further testing will be required to make that determination. For these reasons, we are motivated to further develop thermodynamic integration-based model evidence estimation methods.

### 1.2. Thermodynamic Integral Derivation

In this section, we derive the thermodynamic integral form of the model evidence integral. From Bayes’ theorem, for model M, data vector D, model parameter vector Θ, and prior information *I*, we have

(1) p(D|M,I)=∫p(D|Θ,M,I)p(Θ|M,I)dΘ.

Now, introduce an inverse temperature parameter, β, that controls how much the likelihood influences an evidence related function Z(β):

(2) $$Z\left(\beta \right)=\int {\left[p(D|\Theta ,M,I)\right]}^{\beta }p\left(\Theta |M,I\right)\;d\Theta .$$

If β is 0, the integrand of Equation (2) is simply the prior. If β is 1, Equation (2) is the standard evidence integral, as in Equation (1).

Differentiate the log of the evidence with respect to β using the chain rule,

(3) $$\frac{d}{d\beta }\log Z\left(\beta \right)=\frac{1}{Z(\beta )}\frac{d}{d\beta }Z\left(\beta \right).$$

Define a function we will call the energy,

(4) $$E_{L}\left(\Theta \right)=-\log p\left(D|\Theta ,M,I\right).$$

Evaluate the derivative on the right-hand side of Equation (3), substituting in the definition of the energy Equation (4),

(5) $$\begin{array}{cc} \frac{d}{d\beta }\log Z(\beta ) & =\frac{1}{Z(\beta )}\int \frac{d}{d\beta }\exp\left[-\beta E_{L}\left(\Theta \right)\right]p\left(\Theta |M,I\right)\;d\Theta \;, \end{array}$$

(6) $$\begin{array}{cc} \; & =\int -E_{L}\left(\Theta \right)\frac{\exp\left[-\beta E_{L}\left(\Theta \right)\right]p\left(\Theta |M,I\right)}{Z(\beta )}\;d\Theta \;, \end{array}$$

(7) $$\begin{array}{cc} \; & =-\int E_{L}\left(\Theta \right)p\left(\Theta |M,D,\beta ,I\right)\;d\Theta \;. \end{array}$$

Equation (7) is the negative expectation of the energy with respect to the parameter posterior conditioned on β,

(8) $$\frac{d}{d\beta }\log Z\left(\beta \right)=-{\langle E_{L}\left(\Theta \right)\rangle }_{\beta }\;.$$

To obtain the log-evidence, integrate Equation (8) over the domain of β,

(9) $$\log p\left(D|M,I\right)=-\int_{0}^{1}{\langle E_{L}\left(\Theta \right)\rangle }_{\beta }\;d\beta \;.$$

Equation (9) generally cannot be evaluated analytically, nor can the expected energy at each β within the integral be determined analytically. In practice, the following must be done to use Equation (9) effectively:

- use MCMC to estimate the expected energy at each value of β,
- develop a fixed schedule for β or employ an adaptive strategy to choose optimal stepped values of β,
- and compute the quadrature estimate of the integral.

### 1.3. Outline

The remainder of the article is organized as follows. Section 2 describes the general adaptive annealing with the importance sampling process our proposed method is built upon. Section 3 details how we represent model parameters within our proposed method. Section 4 describes the existing binary slice sampling-based thermodynamic integration methods that our proposed method is inspired by. Section 5 lays out our proposed HMC-based thermodynamic integration method. Section 6 enumerates four groups of example problems that were solved using both the existing BSS-based methods, and our proposed HMC-based method and presents results for each problem from each method. Section 7 concludes the article.

## 2. Adaptive Annealing and Importance Sampling

Much of the thermodynamic integration literature, e.g., [9,10], describes methods that use fixed annealing schedules. That is, all of the values of β are decided before any sampling is done. For problems where the distributions involved are not overly complex, this fixed schedule TI works well. However, for problems in which the distributions are based on physical models, are multi-modal, have correlations in the parameters, or have pronounced curving degeneracies, using a fixed schedule can lead to significant error in the log-evidence estimate. Signal detection problems tend to result in complex distributions like this, so to accommodate these distributions, an adaptive temperature annealing schedule can be used. Goggans and Chi [2] lay out a general procedure for deploying a thermodynamic integration method with adaptive annealing, which is described below:

1. Start at β=0 where p(Θ|M,D,β,I)=p(Θ|M,I), and draw *C* samples from this distribution (the prior).
2. Compute the Monte Carlo estimator for the expected energy at the current β,
  (10) $${\langle E_{L}\left(\Theta \right)\rangle }_{\beta }\approx \frac{1}{N}\sum_{t=1}^{C}E_{L}\left(\Theta_{t}\right)\;,$$
  where $\Theta_{t}$ is the current position of the *t*-th Markov chain.
3. Increment β by $\Delta \beta_{i}$, where
  (11) $$\Delta \beta_{i}=\frac{\log\frac{\max w_{j}}{\min w_{j}}}{\max E_{L}\left(\Theta_{i}\right)-\min E_{L}\left(\Theta_{i}\right)},$$
  *j* is the index on the chains, $w_{j}$ is the weight associated with chain *j*, and
  (12) $$w_{j}=\exp\left[-\Delta \beta_{i}E_{L}\left(\Theta_{j}\right)\right].$$
4. Re-sample the population of samples using importance sampling.
5. Use MCMC to refresh the current population of samples. This yields a more accurate sampling of the distribution at the current temperature. This step can be easily parallelized, as each sample’s position can be shifted independently of the others.
6. Return to step 2 and continue until $\beta_{i}$ reaches 1.
7. Estimate Equation (9) using quadrature and the expected energy estimates built up using Equation (10).

### 2.1. Importance Sampling with Re-Sampling

The importance sampling with re-sampling technique used in this method is described in [2] and follows Liu et al. [14]. Once a new value of β is chosen according to the adaptive annealing procedure, importance sampling with re-sampling is used to sample the distribution under the new value of β. The importance weights used for re-sampling are

(13) $$w_{j}=\frac{p(\Theta |M,D,\beta_{i+1},I)}{p(\Theta |M,D,\beta_{i},I)}=\exp\left(-\Delta \beta_{i}E_{L}\left(\Theta_{j}\right)\right),$$

which are then normalized,

(14) $$W_{j}=J\frac{w_{j}}{\sum_{j=1}^{J}w_{j}}.$$

The Monte Carlo approximation of the posterior distribution under the new β is then

(15) $$p\left(\Theta |M,D,\beta_{i+1},I\right)\approx \frac{1}{J}\sum_{j=1}^{J}W_{j}\delta \left(\Theta -\Theta_{j}\right).$$

At this point, the current population of samples needs to be re-sampled according to Equation (15). In order to determine the number of each sample that should be kept, the following calculation is used:

(16) $$N_{j}=\sum_{k=0}^{J-1}\left[U\left(u+k-\sum_{i=1}^{j-1}W_{i}\right)-U\left(u+k-\sum_{i=1}^{j}W_{i}\right)\right],$$

where *u* is a uniform random variate on [0,1], and U is the unit step function. If $N_{j}=0$ for a particular sample, that sample is removed. If $N_{j}>1$ for a particular sample, that sample is replicated. If N=1 for a particular sample, that sample is simply kept with no change. A pseudo-code implementation of importance sampling with re-sampling is shown in Algorithm 1.

**Algorithm 1** Importance sampling with re-sampling  1: **function**
ImportanceSampling($w,\alpha ,E^{*},C$)  2:   Sort *w*, α, and $E^{*}$ by *w*  3:   w←(C/∑w)w  4:   u←RAND(0,1)  5:   $w_{old}\leftarrow w$  6:   **for**
i←1,C
**do**  7:     $w_{i}\leftarrow \sum_{k}^{i}w_{old,i}$  8:   **end for**  9:   j←010:   q←−111:   **for**
m←1,C
**do**12:     **while**
$w_{m}>u$
**do**13:       $\alpha_{j}\leftarrow \alpha_{m}$14:       $E_{j}^{*}\leftarrow E_{m}^{*}$15:       q←m16:       u←u+117:       j←j+118:     **end while**19:   **end for**20: **end function**

The importance sampling with re-sampling processes by nature can only replicate or remove existing samples. In order to accurately sample the posterior distribution, these samples need to be refreshed periodically, with their current positions serving as starting points. In the first method, a combination of binary slice sampling and leapfrog sampling accomplish this requirement. In the second method, the No U-Turn Sampler is used instead.

### 2.2. Adaptive Annealing

In step 3, a new value of β is chosen. The importance weights in Equation (13) depend on the change in the value of β, and we would like the weights to be set such that, on average, one sample is discarded and replaced at each temperature. The β update Equation (11) is designed with this goal in mind.

The ratio $W=\frac{\max w_{j}}{\min w_{j}}$ in Equation (11) is a constant set by the user. This constant is the desired ratio of the maximum importance weight in the sample population to the minimum importance weight. As long as this ratio is slightly greater than 1 (e.g., 1.05), the importance sampling process will usually discard and replace no more than one sample per temperature, as is the goal. If the distribution being sampled is not particularly challenging, higher values for the ratio constant can be used for a significant decrease in run time.

The denominator in Equation (11) allows the change in temperature to be controlled by the conditions encountered by the sampler. If the maximum energy and minimum energy are close, it is likely that we are sampling the distribution effectively, and β can be changed by a larger amount without disrupting the overall sampling. However, if the values are far apart, the implication is that we are sampling a more difficult portion of the distribution, and that a more gradual change in β will better serve the overall sampling.

## 3. Representing the Model Parameters

Users of the techniques described in this article may wish to evaluate the probabilities for any of a wide variety of mathematical models. The parameters of these models can be assigned any (proper) prior distribution, as the user deems necessary. Ultimately, the techniques developed in this article need to be able to sample model parameter spaces according to these prior distributions. While this challenge can be met in several ways, we take an approach that involves introducing a parameter transformation step into the energy function calculation.

Within the TI-based methods proposed here, parameters are always represented as either integer values on $\left[0,2^{B}\right]$ (for TI with Binary Slice Sampling) or floating point values on the interval [0,1] (for TI with Stan), each with an independent uniform prior distribution. A parameter transformation routine must be included in the energy calculation function that maps these internal parameter representations to values with the correct prior probabilities for computing the energy. If the desired true prior distribution is also uniform, the parameter transformation amounts to a simple scaling operation. Other prior distributions, such as Gaussian distributions, have similar straightforward mapping functions.

In the case that a specialized mapping function is not available, the prior CDF may be used in all cases to perform the mapping through a process known as inverse transform sampling. Let *u* be a variate drawn from Uniform(0,1), let $F_{X}\left(x\right)$ be the CDF for a prior distribution $f_{X}\left(x\right)$, and let $F_{X}^{-1}\left(x\right)$ be the functional inverse of the prior CDF. $F_{X}^{-1}\left(u\right)$ transforms the uniform variate into a variate drawn from $f_{X}\left(x\right)$.

## 4. Thermodynamic Integration with Binary Slice Sampling

Goggans and Chi [2] do not specify how to carry out step 5, the refreshing of the population of samples, but their reference implementation takes inspiration from BayeSys by Skilling [3]. While BayeSys uses several different MCMC “engines” to carry out its sampling, the reference implementation of Goggans and Chi [2] uses just two: binary slice sampling and leapfrog sampling. A pseudo-code listing of this procedure is shown in Algorithm 2.

**Algorithm 2** Thermodynamic integration with binary slice sampling  1: **procedure** TI(P,M,S,N,C,B,W,data)  2:   **Inputs**: *P*–Number of parameters, *M*–Number of chains steps, *S*–Number of slice sampling steps, *N*–New origin probability, *C*–Number of chains, *B*–Number of bits per parameter, *W*–Ratio to control adaptive annealing, data–Data  3:   **for**
m←1,C
**do**  4:     $X\leftarrow R\mathrm{AND}I\mathrm{NT}(0,2^{PB}-1)$  5:     $\alpha^{m}\leftarrow L\mathrm{INE}TOA\mathrm{XES}\left(X,B,P\right)$  6:     $E_{m}^{*}\leftarrow E\mathrm{NERGY}\left(\alpha^{m},data\right)$  7:   **end for**  8:   i←1  9:   Compute ${\langle E^{*}\rangle }_{i}$10:   $\beta_{1}\leftarrow \min\left\{\log\left(W\right)/\left[\max\left(E^{*}\right)-\min\left(E^{*}\right)\right],1\right\}$11:   $w\leftarrow \exp(-\beta_{1}E^{*})$12:   $I\mathrm{MPORTANCE}S\mathrm{AMPLING}(w,\alpha ,E^{*},C)$13:   **while**
$\beta_{i}>0$ and $\beta_{i}<1$
**do**14:     **for**
i←1,M
**do**15:       **for**
m←1,C
**do**16:         $B\mathrm{INARY}S\mathrm{LICE}S\mathrm{AMPLING}(\alpha^{m},E_{m}^{*},B,C,P,S,N,\beta_{i},data)$17:       **end for**18:       $L\mathrm{EAP}F\mathrm{ROG}(\alpha ,E^{*},B,C,P,data)$19:     **end for**20:     i←i+121:     $\Delta \beta \leftarrow \log\left(W\right)/[\max\left(E^{*}\right)-\min\left(E^{*}\right)]$22:     $\beta_{i}\leftarrow \min\left(\beta_{i-1}+\Delta \beta ,1\right)$23:     **if**
$\beta_{i-1}+\Delta \beta >1$
**then**24:       $\Delta \beta \leftarrow 1-\beta_{i-1}$25:     **end if**26:     $w\leftarrow \exp(-\Delta \beta E^{*})$27:     $I\mathrm{MPORTANCE}S\mathrm{AMPLING}(w,\alpha ,E^{*},C)$28:   **end while**29:   Estimate Equation (9) using trapezoid rule and $\left\{\beta_{i}\right\}$ and $\left\{{\langle E^{*}\rangle }_{i}\right\}$30: **end procedure**

Binary slice sampling [15] is an integer-based adaptation of slice sampling by Neal [16]. Slice sampling provides a procedure for sampling distribution f(x). Its procedure for moving from point $x_{0}\in R^{n}$ to another point $x_{1}\in R^{n}$ in a way that maintains detailed balance under distribution f(x) is described below.

A new variable *y* is drawn uniformly from the interval $\left(0,f\left(x_{0}\right)\right)$. The portions of the distribution f(x) that are greater than this value *y* are considered part of the “slice” to be sampled. A stepping-out procedure if performed from $x_{0}$ to find points that are beyond the edges of the slice, then a stepping-in procedure is performed to find bounds that contain all or most of the slice. Once these bounds are obtained, the area defined is sampled uniformly to find the new point, $x_{1}$. This procedure is straightforward only in one dimension, though N-dimensional extensions do exist.

Skilling and MacKay [15]’s binary slice sampling follows this procedure, but it uses integer values for *x* and bit operations to perform the stepping maneuvers and random sampling. A pseudo-code implementation of binary slice sampling is shown in Algorithm 3.

**Algorithm 3** Binary slice sampling  1: **function**
BinarySliceSampling($\alpha ,E^{*},B_{in},C,P,S,N,\beta ,data$)  2:   $B\leftarrow 2^{PB_{in}}$  3:   $X_{orig}\leftarrow R\mathrm{AND}I\mathrm{NT}\left(0,B\right)$  4:   $\alpha_{orig}\leftarrow L\mathrm{INE}TOA\mathrm{XES}\left(X_{orig},B_{in},P\right)$  5:   $\alpha_{i}\leftarrow \alpha$  6:   **for**
i←1,S
**do**  7:     **if**
RAND(0,1)<N
**then**  8:       $X_{orig}\leftarrow R\mathrm{AND}I\mathrm{NT}\left(0,B\right)$  9:       $\alpha_{orig}\leftarrow L\mathrm{INE}TOA\mathrm{XES}\left(X_{orig},B_{in},P\right)$10:     **end if**11:     $\alpha_{i}\leftarrow \left(\alpha_{i}-\alpha_{orig}\right)\;\mathrm{mod}\;2^{B_{in}}$12:     $X\leftarrow A\mathrm{XES}TOL\mathrm{INE}(\alpha_{i},B_{in},P)$13:     U←RANDINT(0,B)14:     y←βENERGY(α,data)−log(RAND(0,1))15:     $l\leftarrow PB_{in}$16:     $E^{*\prime }\leftarrow \infty$17:     **while**
$\beta E^{*\prime }>y$ and l>1
**do**18:       $N\leftarrow R\mathrm{AND}I\mathrm{NT}(0,2^{l})$19:       $X^{\prime }\leftarrow \left(\left\{\left[\left(X-U\right)\;\mathrm{mod}\;B\right]⊕N\right\}+U\right)\;\mathrm{mod}\;B$20:       $\alpha_{i}\leftarrow L\mathrm{INE}TOA\mathrm{XES}\left(X^{\prime },B_{in},P\right)$21:       $\alpha_{i}\leftarrow \left(\alpha_{i}+\alpha_{orig}\right)\;\mathrm{mod}\;2^{B_{in}}$22:       $E^{*\prime }\leftarrow E\mathrm{NERGY}\left(\alpha_{i},data\right)$23:       l←l−P24:     **end while**25:     $\alpha \leftarrow \alpha_{i}$26:   **end for**27: **end function**

This implementation uses a space-filling curve to map multi-dimensional coordinates to a single large integer value, allowing binary slice sampling to be used to sample multi-dimensional distributions without any modification.

It should be noted that the reference TI implementation by Goggans and Chi [2], BayeSys by Skilling [3], and our TI-based methods all omit the stepping-out portion of slice sampling. Instead of first stepping out, our TI-based methods begin by setting the slice width as the maximum range allowed by the prior and step in from there.

This TI implementation also uses leapfrog sampling to help shuffle the sample population more effectively. Leapfrog sampling uses samples’ neighbors to generate new trial positions, and it works as follows. The population is sorted according to each sample’s line (Hilbert or Z-order) index. For each sample in the sorted list, the left and right neighbors are determined. The midpoint between the two neighbor samples in real parameter space is found, and the sample is reflected across that midpoint. If the new position is still between the left and right neighbors, a Metropolis acceptance test is carried out, and the new position is either accepted or rejected. A pseudo-code implementation of the leapfrog sampling method is shown in Algorithm 4.

**Algorithm 4** Leapfrog sampling function  1: **procedure**
Leapfrog($\alpha ,E^{*},B,C,P,data$)  2:   **for**
m←1,C
**do**  3:     $X_{m}\leftarrow A\mathrm{XES}TOL\mathrm{INE}\left(\alpha_{m},B,P\right)$  4:   **end for**  5:   Sort *X*  6:   **for**
m←1,C
**do**  7:     $\alpha_{m}\leftarrow L\mathrm{INE}TOA\mathrm{XES}\left(X_{m},B,P\right)$  8:     $E_{m}^{*}\leftarrow E\mathrm{NERGY}\left(\alpha_{m},data\right)$  9:   **end for**10:   **for**
m←1,C
**do**11:     $\alpha cur\leftarrow \alpha_{m}$12:     **if**
m=1
**then**13:       $l\leftarrow \alpha_{C}$14:     **else**15:       $l\leftarrow \alpha_{m-1}$16:     **end if**17:     **if**
m=C
**then**18:       $r\leftarrow \alpha_{1}$19:     **else**20:       $r\leftarrow \alpha_{m+1}$21:     **end if**22:     Xl←AXESTOLINE(l,B,P)23:     Xr←AXESTOLINE(r,B,P)24:     $\alpha new\leftarrow \left(l+r-\alpha cur\right)\;\mathrm{mod}\;2^{B}$25:     Xnew←AXESTOLINE(αnew,B,P)26:     **if** (m=1 and (Xnew>Xl or Xnew<Xr)) or (m=C and (Xnew>Xl or Xnew<Xr)) or (m>1 and m<C and Xnew>Xl and Xnew<Xr
**then**27:       Enew←ENERGY(αnew,data)28:       **if**
$Enew<E_{m}^{*}$
**then**29:         $E_{m}^{*}\leftarrow Enew$30:         $\alpha_{m}\leftarrow \alpha new$31:       **else**32:         u←RAND(0,1)33:         **if**
$Enew-E_{m}^{*}<-\log\left(u\right)$
**then**34:           $E_{m}^{*}\leftarrow Enew$35:           $\alpha_{m}\leftarrow \alpha new$36:         **end if**37:       **end if**38:     **end if**39:   **end for**40: **end procedure**

Algorithm 2 allows for several BSS steps per leapfrog step to allow for good mixing.

### 4.1. Space-Filling Curves

A space-filling curve is a continuous and nowhere-differentiable function that maps the unit line to a unit hypercube of arbitrary dimension. When we refer to space-filling curves from here on in this article, we are referring to something related but slightly different: an approximation to a true space-filling curve that takes the form $f:N_{0}^{1}\to N_{0}^{N}$ and maps the one-dimensional natural numbers to the *N*-dimensional natural numbers. This approximation to the space-filling curve allows multidimensional probability distributions to be sampled using one-dimensional sampling techniques, such as binary slice sampling. Specifically, it allows us to evaluate model probabilities for models with multiple parameters by creating a 1-to-1 mapping from the multidimensional parameter space to a one-dimensional integer index. The ideal space-filling curve for this application would have the following properties:

- Locality. Points that are nearby in $N_{0}^{N}$ should be nearby on the curve index in $N_{0}^{1}$ as well. The converse should be true as well.
- Time-efficiency. The algorithms for performing the mapping between parameter space and curve indexes should be time-efficient.
- Bi-directionality. Algorithms should exist for mapping parameter space to curve indexes and from curve indexes to parameter space.

#### 4.1.1. Hilbert Curve

**Figure 1:**
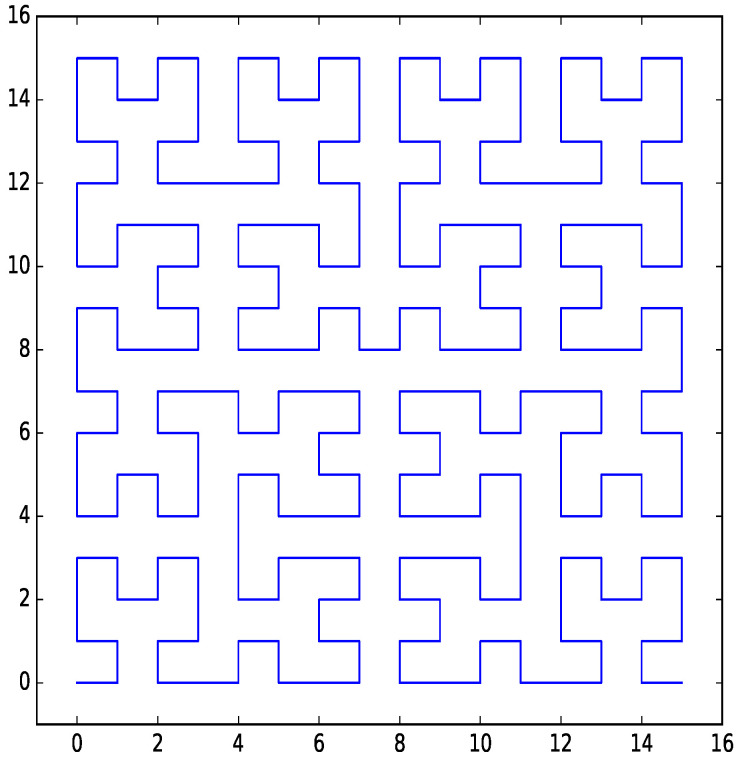
Hilbert curve for two dimensions with four bits per dimension.

The discrete approximation of the Hilbert curve (hereafter referred to simply as the Hilbert curve) [17] (Chapter 2), Ref. [18] is a space-filling curve that has good locality properties. If two indexes are consecutive on the Hilbert curve, the points in parameter space that correspond to them are adjacent. There are also bi-directional transform functions available for the Hilbert curve, and these transform functions can be implemented in a time-efficient way. An example 4-bit per dimension Hilbert curve for a two-dimensional parameter space is shown in Figure 1.

A pseudo-code implementation of the Hilbert curve index-to-parameter transformation is shown in Algorithm 5.

**Algorithm 5** Hilbert curve line-to-axes function  1: **function**
LineToAxes(Line,B,P)  2:   **for**
i←1,P
**do**  3:     $linen_{P-i}\leftarrow (Line\gg \left(B\left(i-1\right)\right)\;\mathrm{mod}\;2^{B}$  4:   **end for**  5:   M←1≪B−1  6:   **for**
i←1,P
**do**  7:     $X_{i}\leftarrow 0$  8:   **end for**  9:   q←0,   p←M10:   **for**
i←1,P
**do**11:     j←M12:     **while**
j>0
**do**13:       **if**
$linen_{i}\wedge j$
**then**14:         X.q←X.q∨p15:       **end if**16:       q←q+117:       **if**
q=n
**then**18:         q←0,   p←p≫119:       **end if**20:       j←j≫121:     **end while**22:   **end for**23:   $t\leftarrow X_{P}\gg 1$24:   **for**
i←P,2
**do**25:     $X_{i}\leftarrow X_{i}⊕X_{i-1}$26:   **end for**27:   $X_{1}\leftarrow X_{1}⊕t$,   M←2≪(B−1),   Q←228:   **while**
Q≠M
**do**29:     P←Q−130:     **for**
i←P,2
**do**31:       **if**
$X_{i}\wedge Q$
**then**32:         $X_{1}\leftarrow X_{1}⊕P$33:       **else**34:         $t\leftarrow (X_{1}⊕X_{i})\wedge P$,   $X_{1}\leftarrow X_{1}⊕t$,   $X_{i}\leftarrow X_{i}⊕t$35:       **end if**36:     **end for**37:     **if**
$X_{1}\wedge Q$
**then**38:       $X_{1}\leftarrow X_{1}⊕P$39:     **end if**40:     Q←Q≪141:   **end while**42:   **return**
*X*43: **end function**

#### 4.1.2. Z-Order Curve

**Figure 2:**
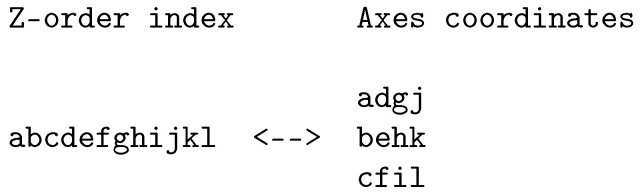
Example of Z-order bit-interleaving for three dimensions with four bits per dimension.

The Z-order curve [17] (Chapter 5), Ref. [19] (also known as the Lebesgue curve or Morton curve) is a space-filling curve that maintains locality somewhat less well than the Hilbert curve but otherwise fulfills our requirements for a space-filling curve. Importantly, its transformation algorithms are faster than those for the Hilbert curve. In order to transform from the N-dimensional parameter space to the one-dimensional, Z-order curve, the bits of the integer coordinates for each dimension are interleaved. If the parameter space has three dimensions and each coordinate axis is represented by a 4-bit integer, the resulting Z-order curve representation will be a 12-bit integer. An example of this bit-interleaving is shown in Figure 2, in which each letter represents a binary digit, demonstrates the bit interleaving described above.

**Figure 3:**
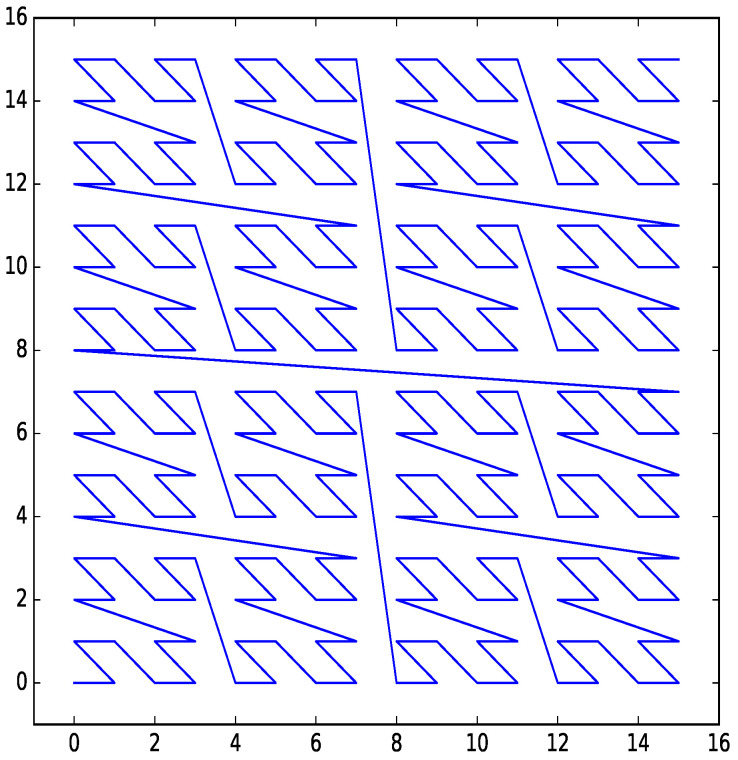
Z-order curve for two dimensions with four bits per dimension.

An example of a Z-order curve for two dimensions with four bits per dimension is shown in Figure 3.

The simple way to perform the Z-order mapping is to loop over the bit and axis indexes and place each bit where it needs to be individually. However, this method does not provide a time complexity improvement over the Hilbert curve transform functions. A cleverer, bitmask-based approach exists, and it is documented in several places online. The most thorough description of an algorithm for generating the necessary bitmasks for arbitrary numbers of dimensions and bits per dimension is given in a Stackoverflow answer by user Gabriel [20]. Gabriel also describes the general method by which the mapping is performed using the bitmasks, but he does not provide algorithms for doing so. The following list outlines the basic procedure for mapping from the Z-order index to axes coordinates:

1. Generate bitmasks based on number of bits *b* and number of parameters *n*.
2. AND the first mask with the Z-order integer to select only every *n* bits.
3. Loop over each mask. For the *i*th mask, XOR the Z-order integer with itself shifted to the right by *i*, then mask the result.
4. Shift the original Z-order integer to the right by 1, then repeat the above from step 2 for each dimension.

A pseudo-code implementation for the bitmask computation function is shown in Algorithm 6, and a pseudo-code implementation for the bitmask-based line-to-axes transformation function is shown in Algorithm 7.

**Algorithm 6** Z-order curve mask computation function  1: **function**
ComputeBitMask(B,P)  2:   P←P−1  3:   **for**
i←1,B
**do**  4:     $bd_{i}\leftarrow \left(i+1\right)P$        ▹ Stored as binary strings, with the leftmost two bits discarded  5:   **end for**  6:   maxLength← length of longest string in bd  7:   moveBits← empty list  8:   **for**
i←1,maxLength
**do**  9:     Append an empty list to moveBits10:     **for**
j←1,P
**do**11:       **if**
$L\mathrm{ENGTH}(bd_{j})\geq i$
**then**12:         **if** The *i*th bit from the end of $bd_{j}$ is 1 **then**13:           Append *j* to $moveBits_{i}$14:         **end if**15:       **end if**16:     **end for**17:   **end for**18:   **for**
i←1,B
**do**19:     $bitPositions_{i}\leftarrow i$20:   **end for**21:   maskOld←(1≪B)−122:   bitmasks← empty list23:   **for**
i←LENGTH(moveBits),1
**do**24:     **if**
$L\mathrm{ENGTH}(moveBits_{i})>0$
**then**25:       shifted←026:       **for**
$bitIdxToMove\in moveBits_{i}$
**do**27:         $shifted\leftarrow shifted\vee (1\ll bitPositions_{bitIdxToMove})$28:         $bitPositions_{bitIdxToMove}\leftarrow bitPositions_{bitIdxToMove}+2^{i}$29:       **end for**30:       nonshifted←¬shifted∧maskOld31:       $shifted\leftarrow shifted\ll 2^{i}$32:       maskNew←shifted∨nonshifted33:       Append maskNew to bitmasks34:       maskOld←maskNew35:     **end if**36:   **end for**37:   **return**
bitmasks38: **end function**

**Algorithm 7** Z-Order curve line-to-axes function  1: **function**
LineToAxes(z,B,P)  2:   **if**
P=1
**then**  3:     **return**
*z*  4:   **end if**  5:   masks←COMPUTEBITMASK(B,P)        ▹ Call only once for each *B* and *P*  6:   Pop final entry from masks list into first  7:   Reverse the masks list  8:   Append 1≪B to masks  9:   minshift←P−110:   **for**
i←1,P
**do**11:     zz←z≫i12:     zz←zz∧first13:     shift←minshift14:     **for**
mask∈masks
**do**15:       zz←(zz⊕(zz≫shift))∧mask16:       shift←shift≪117:     **end for**18:     $\alpha_{i}\leftarrow zz$19:   **end for**20:   **return**
α21: **end function**

### 4.2. Parallel Implementation

In implementations of this binary slice sampling and space-filling curve-based technique, most of the computation time is taken up by the line-to-axes and axes-to-line operations. Because the vast majority of the invocations of these operations occur during the binary slice sampling routine itself, the for loop on lines 15, 16, and 17 in Algorithm 2 is a natural place to parallelize the algorithm. Parallelization at this point allows for much larger sample populations (*C*), which improves the accuracy of the evidence estimate. The TI-BSS example results in Section 6 are produced with a parallel implementation in this vein.

## 5. Thermodynamic Integration with Stan

The evolution of our approach proceeded as follows. John Skilling released the latest version of BayeSys [3] in the late 2000s. This software used an adaptively annealed thermodynamic integration framework combined with binary slice sampling on the Hilbert curve to perform jump-diffusion sampling. This approach is useful for doing model comparison in the case that the model equation can be decomposed into several identical components.

Soon afterward, Paul Goggans and Ying Chi wanted to apply this adaptively-annealed thermodynamic integration framework to a model comparison problem in which the model equation was not separable. They developed a method, TI-BSS-H [2] that used a very similar approach to BayeSys, but rather than using the thermodynamic integration framework to facilitate jump diffusion sampling, their method directly estimated the evidence for a particular model.

When our interest again returned to thermodynamic integration in 2017, we published a method, TI-BSS-Z [19] that replaced the Hilbert curve component of the TI-BSS-H approach with the Z-order curve. This change was made with the goal of increasing the speed of the method. Ultimately, this approach sacrificed too much accuracy for us to use it on a regular basis.

More recently, we have taken a different tack in further developing this approach. We have gone beyond replacing the space-filling curve used for BSS and instead replaced the BSS component all together with a more modern MCMC method. A recent survey of available MCMC tools turned up MC Stan (or simply Stan), developed by Carpenter et al. [4], as the state-of-the-art, general purpose MCMC method for performing Bayesian parameter estimation.

Stan uses the NUTS [6] as the basis for its sampling functions. NUTS is based on HMC [21], which uses the gradient of the log-likelihood function to more efficiently explore the posterior distribution. NUTS improves upon HMC by automatically choosing optimal values for HMC’s tunable method parameters. NUTS has been shown to sample complex distributions effectively. We sought to build an improved thermodynamic integration implementation by using Stan instead of binary slice sampling and leapfrog sampling to refresh the sample population at each temperature within TI. The result, TI-Stan, is described in this section.

The TI-Stan algorithm is shown in Algorithm 8.

**Algorithm 8** Thermodynamic integration with Stan  1: **procedure** TI(P,S,C,W,data)  2:   **Inputs**: *P*–Number of parameters, *S*–Number of Stan iterations per temperature, *C*–Number of chains, *W*–Ratio to control adaptive annealing, data–Data  3:   **for**
m←1,C
**do**  4:     $X\leftarrow R\mathrm{AND}I\mathrm{NT}(0,2^{PB}-1)$  5:     $\alpha^{m}\leftarrow L\mathrm{INE}TOA\mathrm{XES}\left(X,B,P\right)$  6:     $E_{m}^{*}\leftarrow E\mathrm{NERGY}\left(\alpha^{m},data\right)$  7:   **end for**  8:   i←1  9:   Compute ${\langle E^{*}\rangle }_{i}$10:   $\beta_{1}\leftarrow \min\left\{\log\left(W\right)/\left[\max\left(E^{*}\right)-\min\left(E^{*}\right)\right],1\right\}$11:   $w\leftarrow \exp(-\beta_{1}E^{*})$12:   $I\mathrm{MPORTANCE}S\mathrm{AMPLING}(w,\alpha ,E^{*},C)$13:   **while**
$\beta_{i}>0$ and $\beta_{i}<1$
**do**14:     **for**
m←1,C
**do**15:       $S\mathrm{TAN}S\mathrm{AMPLING}(\alpha^{m},E_{m}^{*},C,P,S,\beta_{i},data)$16:     **end for**17:     i←i+118:     $\Delta \beta \leftarrow \log\left(W\right)/[\max\left(E^{*}\right)-\min\left(E^{*}\right)]$19:     $\beta_{i}\leftarrow \min\left(\beta_{i-1}+\Delta \beta ,1\right)$20:     **if**
$\beta_{i-1}+\Delta \beta >1$
**then**21:       $\Delta \beta \leftarrow 1-\beta_{i-1}$22:     **end if**23:     $w\leftarrow \exp(-\Delta \beta E^{*})$24:     $I\mathrm{MPORTANCE}S\mathrm{AMPLING}(w,\alpha ,E^{*},C)$25:   **end while**26:   Estimate Equation (9) using trapezoid rule and $\left\{\beta_{i}\right\}$ and $\left\{{\langle E^{*}\rangle }_{i}\right\}$27: **end procedure**

Our implementation is in Python, so we made use of the PyStan interface to Stan, developed by the Stan Development Team [5]. Stan defines its own language for defining statistical models, which allows it to efficiently compute the derivatives needed for HMC via automatic differentiation. For a particular problem, it is therefore necessary to write a Stan file that contains the Stan-formatted specification of the model, in addition to the pure-Python energy functions necessary for TI-BSS. Once one is familiar with the simple Stan language, this additional programming cost becomes trivial compared to the time savings achieved by using this method instead of TI-BSS.

## 6. Examples

In this section, we demonstrate the performance of the TI-Stan method using four problems as examples. These include an artificial, highly multi-modal likelihood function, a simulated spectrum analysis problem, a twin Gaussian shell problem in 20 and 30 dimensions, and the estimation of the thermodynamic partition function for an ideal gas.

### 6.1. Eggcrate Likelihood

The first example is the “eggcrate” toy problem from [22]. The posterior density in this problem is highly multimodal, so that a large sample population is necessary to estimate the evidence accurately and to sample the entire density.

The eggcrate function has two independent parameters, with joint prior

(17) $$\pi \left(\Theta \right)={\left(\frac{1}{10\pi }\right)}^{2}{𝟙}_{\left[0,10\pi \right]}\left(\Theta_{1}\right){𝟙}_{\left[0,10\pi \right]}\left(\Theta_{2}\right).$$

The likelihood function is

(18) $$L\left(\Theta \right)=\exp\left\{{\left[2+\cos\left(\frac{\Theta_{1}}{2}\right)\cos\left(\frac{\Theta_{2}}{2}\right)\right]}^{5}\right\}.$$

In this case, the log-likelihood is more useful for visualization and the numerical dynamic range:

(19) $$\log L\left(\Theta \right)={\left[2+\cos\left(\frac{\Theta_{1}}{2}\right)\cos\left(\frac{\Theta_{2}}{2}\right)\right]}^{5}.$$

Feroz et al. [22] provide an evidence value of logZ=235.88 using numerical integration over a fine grid. Upon applying Bayes’ theorem, the posterior distribution for the parameters Θ is

(20) $$P\left(\Theta \right)=\frac{\left.\exp\left\{{\left[2+\cos\left(\frac{\Theta_{1}}{2}\right)\cos\left(\frac{\Theta_{2}}{2}\right)\right]}^{5}\right\}\right.\left.{\left(\frac{1}{10\pi }\right)}^{2}{𝟙}_{\left[0,10\pi \right]}\left(\Theta_{1}\right){𝟙}_{\left[0,10\pi \right]}\left(\Theta_{2}\right)\right.}{\exp(235.88)}.$$

### 6.2. Detection of Multiple Stationary Frequencies

In the second example, we want to estimate the number of stationary frequencies present in a signal as well as the value of each frequency. This problem is similar to the problem of multiple stationary frequency estimation in [23] (Chapter 6), with the additional task of determining the number of stationary frequencies present. This example demonstrates the value of the present parallel nested sampling method. Differences among log-evidence values for models containing either the most probable number of frequencies or more tend to be small, meaning that a precise estimate of these log-evidence values is essential for the task of determining the most probable model.

Each stationary frequency (*j*) in the model is determined by three parameters: the in-phase amplitude ($A_{j}$), the quadrature amplitude ($B_{j}$), and the frequency ($f_{j}$). Given *J* stationary frequencies, the model at time step $t_{i}$ takes the following form:

(21) $$g\left(t_{i};\Theta \right)=\sum_{j=1}^{J}A_{j}\cos\left(2\pi f_{j}t_{i}\right)+B_{j}\sin\left(2\pi f_{j}t_{i}\right),$$

where Θ is the parameter vector

$$\Theta ={\left[A_{1}\;B_{1}\;f_{1}\;\cdots \;A_{J}\;B_{J}\;f_{J}\right]}^{T}.$$

For the purposes of this example, the noise variance used to generate the simulated data is known, and we consequently use a Gaussian likelihood function,

(22) $$L\left(\Theta \right)=\prod_{i=1}^{I}\exp\left\{-\frac{{\left[g\left(t_{i};\Theta \right)-d_{i}\right]}^{2}}{2\sigma^{2}}\right\},$$

for *I* simulated data $d_{i}$ and noise variance $\sigma^{2}$. The log-likelihood function is then

(23) $$\log L\left(\Theta \right)=-\sum_{i=1}^{I}\frac{{\left[g\left(t_{i};\Theta \right)-d_{i}\right]}^{2}}{2\sigma^{2}}.$$

Each model parameter is assigned a uniform prior distribution with limits as shown in Table 1.

Our test signal is a sum of two sinusoids, and zero-mean Gaussian noise with variance $\sigma^{2}=0.01$. This signal is sampled at randomly-spaced instants of time, in order to demonstrate that this time-domain method does not require uniform sampling to perform spectrum estimation. Bretthorst [24] demonstrates that the Nyquist critical frequency in the case of nonuniform sampling is $1/2\Delta T^{\prime }$, where $\Delta T^{\prime }$ is the dwell time. The dwell time is not defined for arbitrary-precision time values as used in this example, so we must choose another limiting value. A more conservative limit is given by $1/10\Delta T_{\mathrm{avg}}$, where $\Delta T_{\mathrm{avg}}$ is the average spacing between time steps, 1/64 s. This formulation yields a prior maximum limit of 6.4 Hz, as shown in Table 1. The parameters used to generate the simulated data are shown in Table 2.

**Figure 4:**
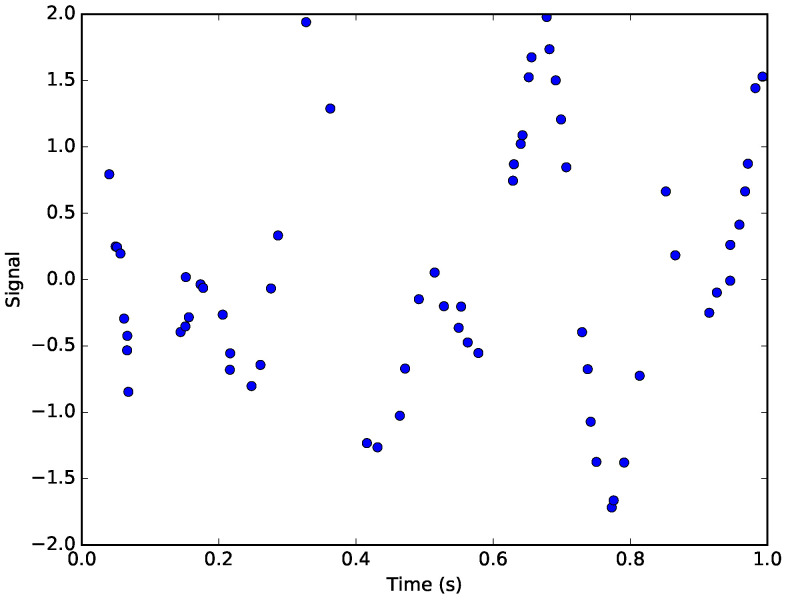
The simulated signal. The points represent the non-uniformly sampled points from the original signal corrupted by Gaussian noise.

The samples from the signal with noise are shown in Figure 4.

### 6.3. Twin Gaussian Shells

The third example is the twin Gaussian shell problem, also from [22]. In [22], the authors present results for this problem in up to 30 dimensions. Handley et al. [25] also use this problem in 100 dimensions to test their algorithm. This problem presents a few interesting challenges to our thermodynamic integration implementation. Because the likelihood takes the form of a thin, curved density whose mass centers on a hyper-spherical shell, exploration of the posterior at high inverse temperature values is difficult. The bimodal nature of the problem also challenges the posterior exploration process. Finally, the examples we explore are high-dimensional to the point that standard numerical integration techniques would be useless.

The likelihood function in the twin Gaussian shells problem takes the form,

(24) $$L\left(\Theta \right)=\frac{1}{\sqrt{2\pi }w_{1}}\exp\left[-\frac{(|\Theta -c_{1}{\left|-r_{1}\right)}^{2}}{2w_{1}^{2}}\right]+\frac{1}{\sqrt{2\pi }w_{2}}\exp\left[-\frac{(|\Theta -c_{2}{\left|-r_{2}\right)}^{2}}{2w_{2}^{2}}\right].$$

**Figure 5:**
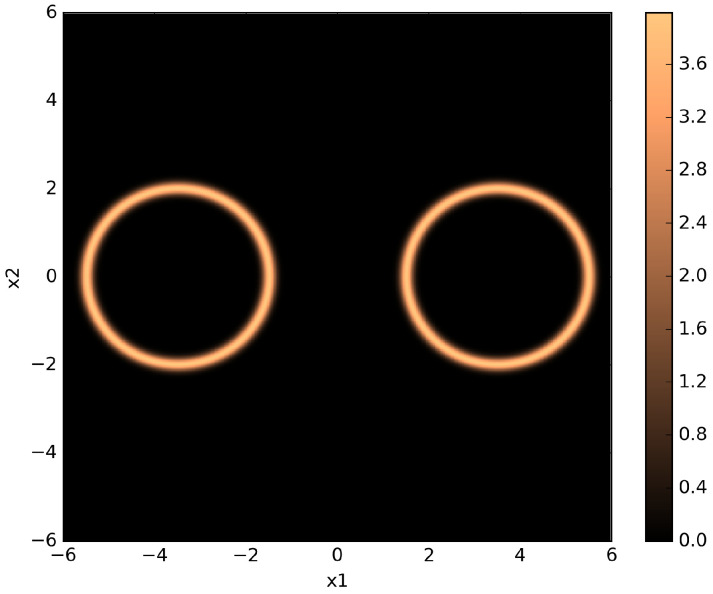
Pseudo-color plot of a two-dimensional twin Gaussian shell with $w_{1}=w_{2}=0.1$, $r_{1}=r_{2}=2$, $c_{1}={\left[-3.5,0\right]}^{T}$, and $c_{2}={\left[3.5,0\right]}^{T}$. The color values correspond to likelihood values.

Following [22], we set the parameters as follows: $w_{1}=w_{2}=0.1$, $r_{1}=r_{2}=2$, $c_{1}={\left[-3.5,0,\cdots ,0\right]}^{T}$, and $c_{2}={\left[3.5,0,\cdots ,0\right]}^{T}$. We use a uniform prior over the hypercube that spans [−6,6] in each dimension. Figure 5 shows a pseudo-color plot of a two-dimensional twin Gaussian shell with parameters and prior range as described previously.

### 6.4. Ideal Gas Partition Function

The final example is inspired by Section 31.3 of [26]. In this example problem, we wish to estimate a function proportional to the thermodynamic partition function of an ideal gas with $N_{p}=N/3$ particles,

(25) $$Z=\int \exp\left[-p^{2}/\left(2\sigma^{2}\right)\right]\;d^{N}p,$$

where *p* is an *N*-dimensional vector of the particles’ momenta, and $\sigma^{2}=mk_{B}T$ is the product of the mass, Boltzmann constant, and temperature. Following [26], we assign a uniform prior distribution to the momenta p with support on an (N−1)-sphere S(R) of radius *R*, set such that the error in the evaluation of the integral is below an acceptable threshold,

(26) $$R=2\sqrt{N}\sigma .$$

The volume of S(R) is

(27) $$V_{N}\left(R\right)=\frac{R^{N}\pi^{N/2}}{\Gamma \left(\frac{N}{2}+1\right)}.$$

Under this prior, we can rewrite the partition function in the form

(28) $$Z=V_{N}\left(R\right)\underset{\tilde{Z}}{\underset{︸}{\int_{S(R)}\exp\left[-x^{2}/\left(2\sigma^{2}\right)\right]p_{0}\left(x\right)\;d^{N}x}},$$

where $p_{0}\left(x\right)$ is the prior, $\exp[-x^{2}/\left(2\sigma^{2}\right)]$ is the likelihood, and $\tilde{Z}$ is the function that we will actually estimate and show results for. The exact value is given by

(29) $$\tilde{Z}=\frac{{\left(2\pi \sigma^{2}\right)}^{N/2}}{V_{N}\left(R\right)}.$$

Substituting Equation (26) into Equation (29), taking the log, and simplifying yields the exact value

(30) $$\log\tilde{Z}=-\frac{N}{2}\log2-\frac{N}{2}\log N+\log\Gamma \left(\frac{N}{2}+1\right).$$

We use Equation (30) to judge the accuracy of our numerical results.

### 6.5. Results

For the first three examples, the settings used for TI-BSS are shown in Table 3, while the settings used for TI-Stan are shown in Table 4. For each example, the user-defined constant W was set to both 1.5 and 2.0. Box-plots are used extensively in this section. In these box-plots, the middle line represents the median value, the box is bounded by the upper and lower quartiles, and the whiskers extend to the range of the data that lies within 1.5 times the inter-quartile range. Any data points past this threshold are plotted as circles.

These results for the first three examples were generated on a Google Cloud (Mountain View, CA, USA) instance with 32 virtual Intel Broadwell CPUs and 28.8 GB of RAM.

#### 6.5.1. Eggcrate Likelihood Results

The true value of the log-evidence for this log-likelihood function is known up to five significant digits:

(31) $$\log Z_{\mathrm{true}}=235.88.$$

**Figure 6:**
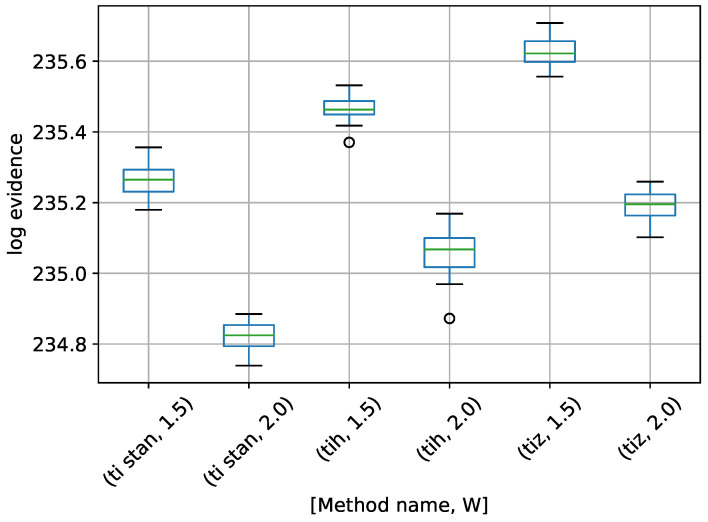
Box-plot of log-evidence for the egg-crate problem for each TI method. TI-Stan with W=xx is denoted by ti stan, xx; TI-BSS-H with W=xx is denoted by tih, xx; and TI-BSS-Z with W=xx is denoted by tiz, xx.

A box-plot summarizing the log-evidence estimates over 20 runs each for the TI-Stan, TI-BSS-H, and TI-BSS-Z methods and for each value of *W* is shown in Figure 6.

Figure 6 demonstrates that all of the TI methods at these settings underestimate the log-evidence, with TI-BSS-Z with W=1.5 coming the closest. Likely a value of *W* closer to 1 would yield better results. It is also apparent that the precision in these results do not correlate with the accuracy, suggesting that, for problems with unknown answers, high precision over multiple runs should not be interpreted as a proxy for accuracy.

**Figure 7:**
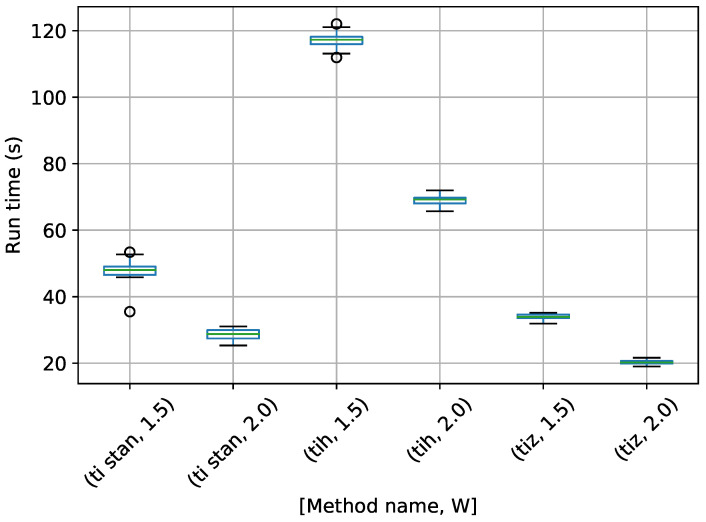
Box-plot of run time in seconds for the egg-crate problem for each TI method. TI-Stan with W=xx is denoted by ti stan, xx; TI-BSS-H with W=xx is denoted by tih, xx; and TI-BSS-Z with W=xx is denoted by tiz, xx.

A box-plot summarizing the run times over 20 runs each for the TI methods is shown in Figure 7.

Figure 7 shows that each method speeds up considerably with a higher value of *W*. TI-BSS-H took the most time here, with TI-Stan and TI-BSS-Z running much more quickly.

#### 6.5.2. Twin Gaussian Shells’ Results

This problem is run in 10 dimensions, 30 dimensions, and 100 dimensions. For the case of 10 dimensions, results are presented for all TI-BSS-H and TI-Stan, but not for TI-BSS-Z. For both values of *W*, TI-BSS-Z ended early in all its runs, and its estimate of the log-evidence is unusably inaccurate. This likely occurred because the Z-order curve sacrifices some locality for the sake of speed compared to the Hilbert curve. Problems due to the reduced locality likely compound in the case of higher dimensionality. For the cases of 30 and 100 dimensions, results are presented only for TI-Stan because a run of TI-BSS-H took too much time for it to be feasible to complete multiple runs. The Hilbert curve calculations become infeasibly time-consuming in high dimensions.

##### 10-Dimensional Twin Gaussian Shells

**Figure 8:**
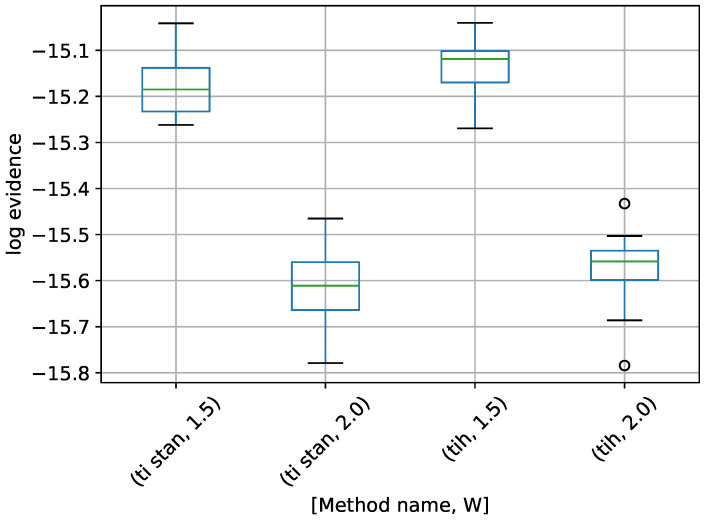
Box-plot of log-evidence for the 10-D twin Gaussian shell problem for TI-Stan and TI-BSS-H. TI-Stan with W=xx is denoted by ti stan, xx and TI-BSS-H with W=xx is denoted by tih, xx.

We start with the 10-dimensional variant. A box-plot summarizing the log-evidence estimates over 20 runs each for TI-Stan and TI-BSS-H and for each value of *W* is shown in Figure 8.

From [22], the analytical log-evidence for this distribution is −14.59. None of the configurations tested actually reached that value, but the runs using W=1.5 got closest, suggesting that a value of *W* closer to 1 would perhaps approach the correct value.

**Figure 9:**
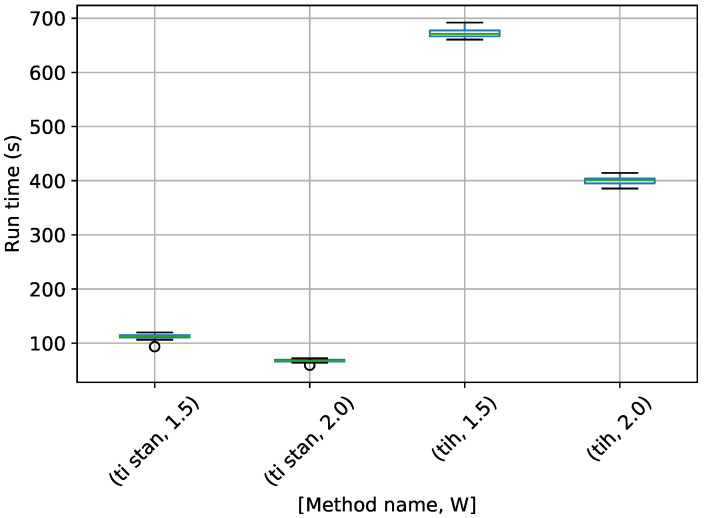
Box-plot of run time in seconds for the 10-D twin Gaussian shell problem for TI-Stan and TI-BSS-H. TI-Stan with W=xx is denoted by ti stan, xx and TI-BSS-H with W=xx is denoted by tih, xx.

A box-plot summarizing the run times over 20 runs each for the TI methods is shown in Figure 9.

Figure 9 shows the same trend with *W* as in the egg-crate problem, i.e., that the run time drastically increases as *W* approaches 1. It also shows that TI-BSS-H takes about six times longer, on average, than TI-Stan to compute its estimate of the log-evidence. According to Figure 8, the two methods have comparable accuracy and precision, so this difference in run time illustrates the difficulty the Hilbert curve-based method has with distributions of high dimension.

##### 30-Dimensional Twin Gaussian Shells

**Figure 10:**
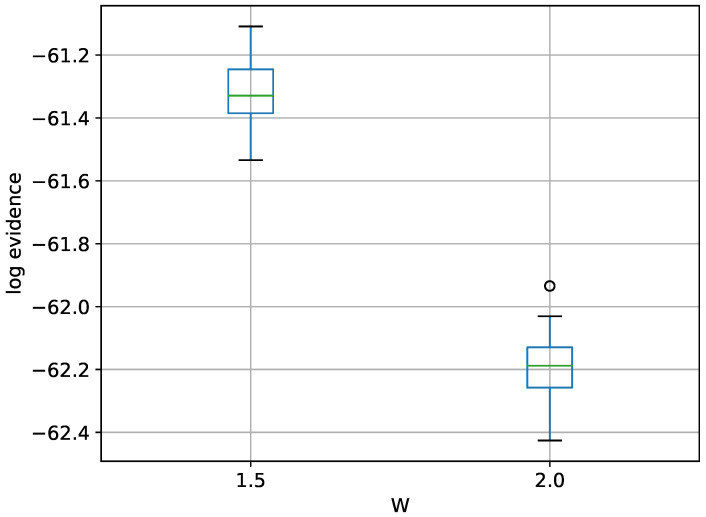
Box-plot of log-evidence for the 30-D twin Gaussian shell problem for TI-Stan.

Regarding the 30-dimensional variant, the limitations of TI-BSS become even more apparent, as one run could not finish in a reasonable amount of time. TI-Stan, however, has no problem with the 30-D variant. A box-plot summarizing the log-evidence estimates over 20 runs each for TI-Stan at two values of *W* is shown in Figure 10.

Again from [22], the analytical log-evidence value is −60.13. Neither value of *W* allows TI-Stan to come within 1 unit of the correct answer here, and the gap is actually slightly larger here than in the 10-D variant. Again, a smaller value of *W* would probably be helpful here.

**Figure 11:**
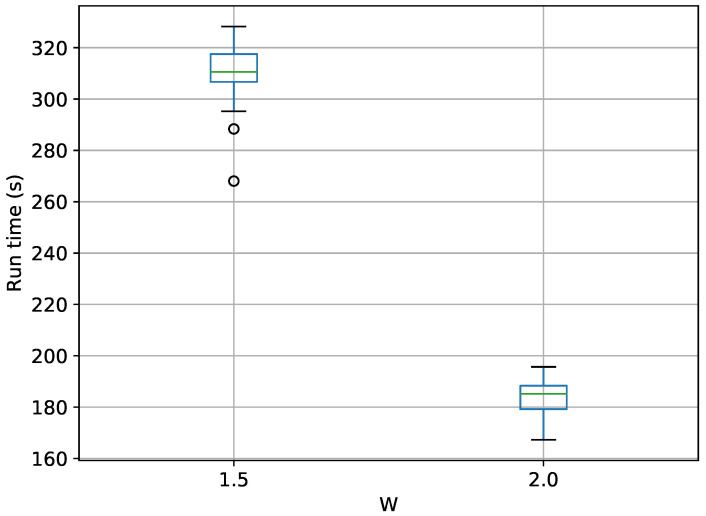
Box-plot of run time in seconds for the 30-D twin Gaussian shell problem for TI-Stan.

A box-plot summarizing the run times over 20 runs each for TI-Stan is shown in Figure 11.

The common trend in run time vs *W* is observed here as well. TI-Stan takes about three times as long on average to compute log-evidence values in the 30-D case compared to the 10-D case, suggesting a possible linear relationship between run time and the number of dimensions for this problem.

##### 100-Dimensional Twin Gaussian Shells

**Figure 12:**
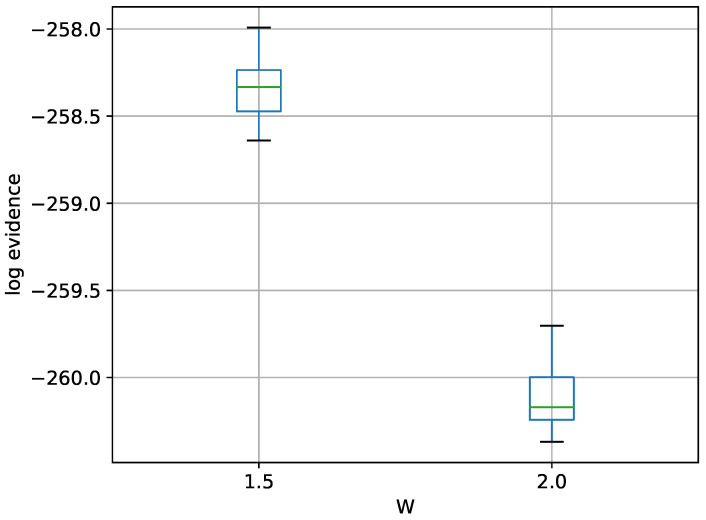
Box-plot of log-evidence for the 100-D twin Gaussian shell problem for TI-Stan.

A box-plot summarizing the log-evidence estimates over 20 runs each for TI-Stan at two values of *W* is shown in Figure 12.

There is no analytical value available in the literature for this variant of the problem, but the result here seems believable. If it follows the trend of the previous examples, the log-evidence has been underestimated by some margin.

**Figure 13:**
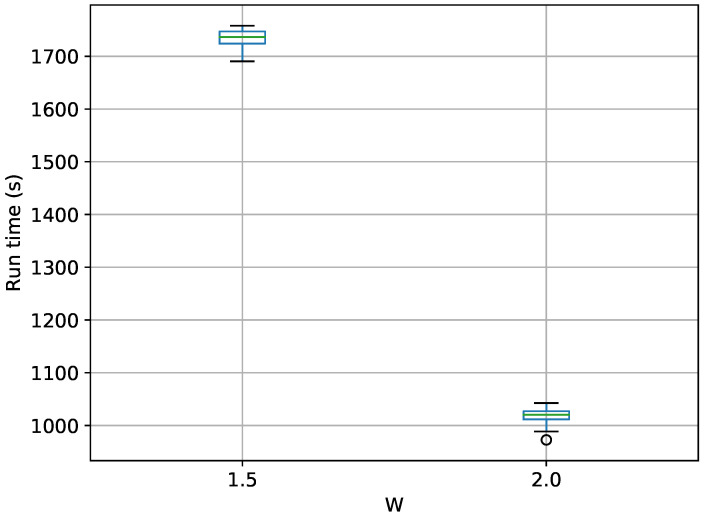
Box-plot of run time in seconds for the 100-D twin Gaussian shell problem for TI-Stan.

A box-plot summarizing the run times over 20 runs each for TI-Stan is shown in Figure 13.

The results in Figure 13 disprove the hypothesis that the run time is related linearly to the number of parameters in this problem. The established relationship between run time and *W* continues here. This example is only a toy problem, but these results suggest that TI-Stan could be useful in problems with actual data and very high-dimensional models.

#### 6.5.3. Detection of Multiple Stationary Frequencies’ Results

**Figure 14:**
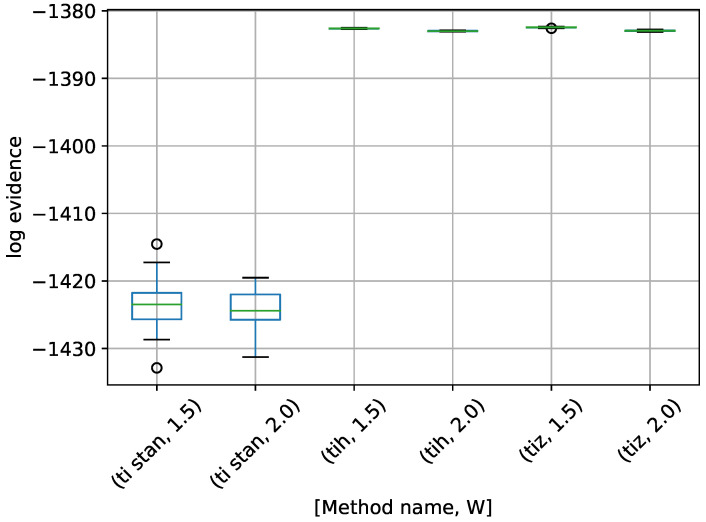
Box-plot of log-evidence for the one stationary frequency model for TI-Stan, TI-BSS-H, and TI-BSS-Z, for two values of *W*. TI-Stan with W=xx is denoted by ti stan, xx; TI-BSS-H with W=xx is denoted by tih, xx; and TI-BSS-Z with W=xx is denoted by tiz, xx.

**Figure 15:**
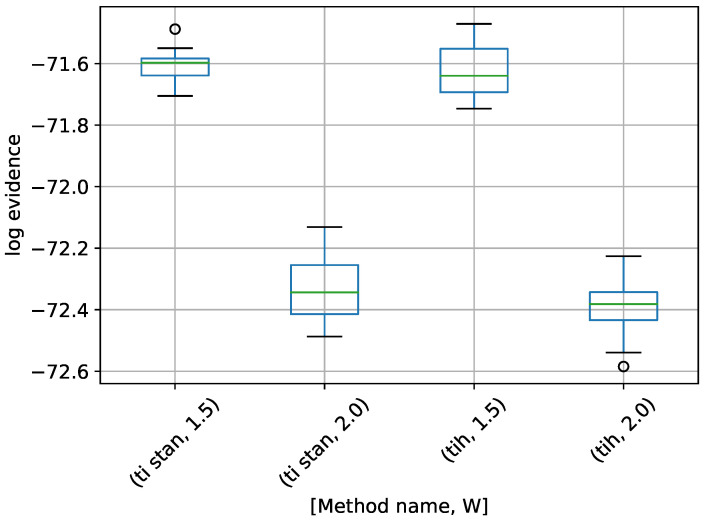
Box-plot of log-evidence for the two stationary frequency model for TI-Stan and TI-BSS-H, for two values of *W*. TI-Stan with W=xx is denoted by ti stan, xx and TI-BSS-H with W=xx is denoted by tih, xx.

**Figure 16:**
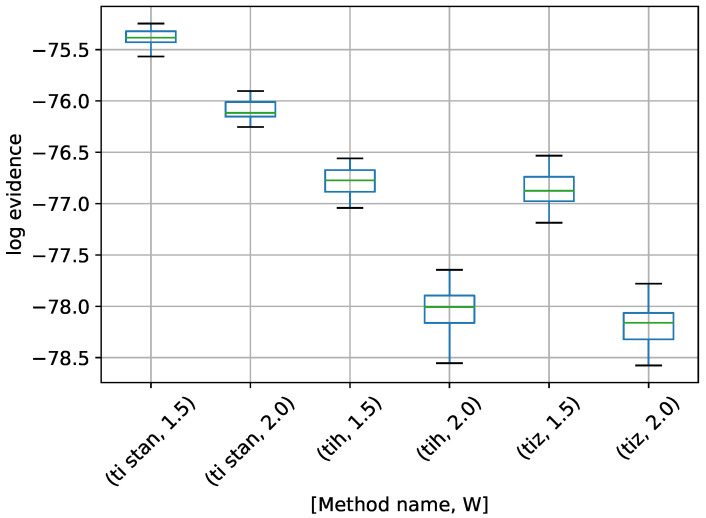
Box-plot of log-evidence for the three stationary frequency model for TI-Stan, TI-BSS-H, and TI-BSS-Z, for two values of *W*. TI-Stan with W=xx is denoted by ti stan, xx; TI-BSS-H with W=xx is denoted by tih, xx; and TI-BSS-Z with W=xx is denoted by tiz, xx.

The final example problem is the multiple stationary frequencies detection problem described in Section 6.2. Box-plots of log-evidence values for a model assuming one, two, and three frequencies present are shown in Figure 14, Figure 15 and Figure 16. For the models with one and three frequencies present, results are shown for TI-Stan, TI-BSS-H, and TI-BSS-Z. For the model with two frequencies present (the model also used to generate the test signal), results for TI-BSS-Z are not shown. As in the Gaussian shell problems, TI-BSS-Z ended early here and did not arrive at a reasonable result. Here, as well, the problem likely lies in the decreased locality of the Z-order curve when compared with the Hilbert curve. In addition, when evaluating the model used to generate the original data (in other words, the “true” model), the likelihood function will be sharper than when evaluating the other candidate models. This, combined with the decreased locality of the Z-order curve, likely led to its failure in this case.

There are no analytical log-evidence values available for this example. We argue that a method is successful if the model used to generate the data clearly has the highest log-evidence, with a good margin between it and the log-evidence for the other models. There is some significant disagreement among the various methods for the “wrong” models (those with one and three frequencies), but the methods are in much closer agreement for the two-frequency model. For TI-Stan and TI-BSS-H and for both values of *W*, the two-frequency model is clearly the maximum-log-evidence choice. Even with the variations in the runs, the results do not overlap at any point from model to model, and the closest model-to-model margins are all greater than 2.3, which corresponds to odds of 10.

**Figure 17:**
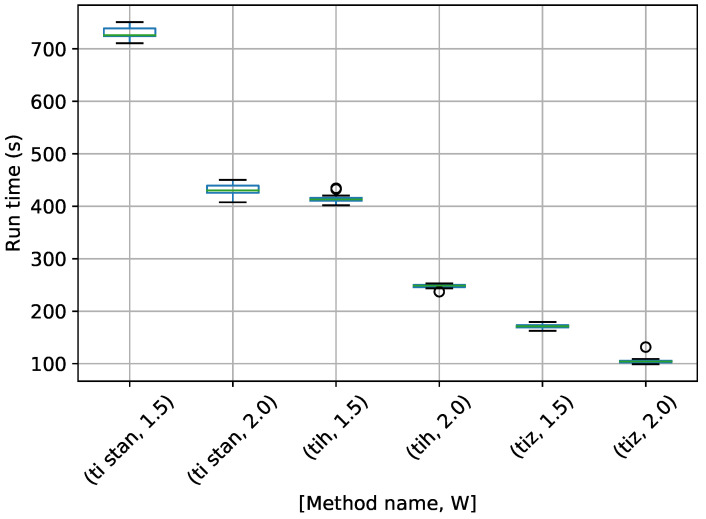
Box-plot of run time for the J=1 stationary frequency model for TI-Stan, TI-BSS-H, and TI-BSS-Z, for two values of *W*. TI-Stan with W=xx is denoted by ti stan, xx; TI-BSS-H with W=xx is denoted by tih, xx; and TI-BSS-Z with W=xx is denoted by tiz, xx.

**Figure 18:**
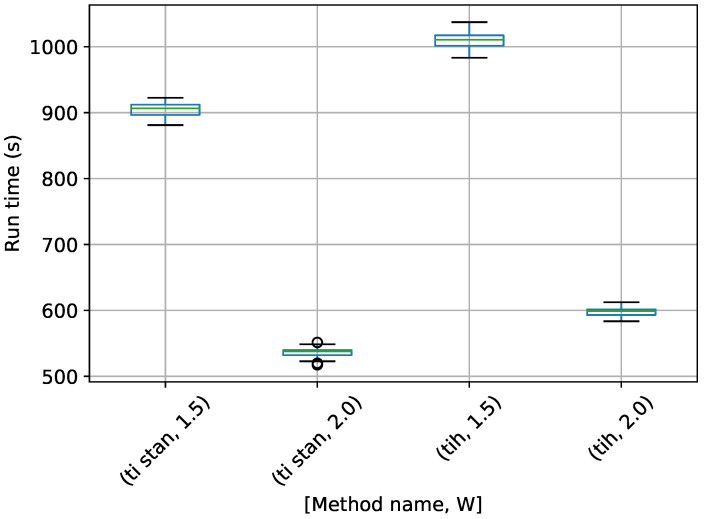
Box-plot of run time for the J=2 stationary frequency model for TI-Stan and TI-BSS-H, for two values of *W*. TI-Stan with W=xx is denoted by ti stan, xx; and TI-BSS-H with W=xx is denoted by tih, xx.

**Figure 19:**
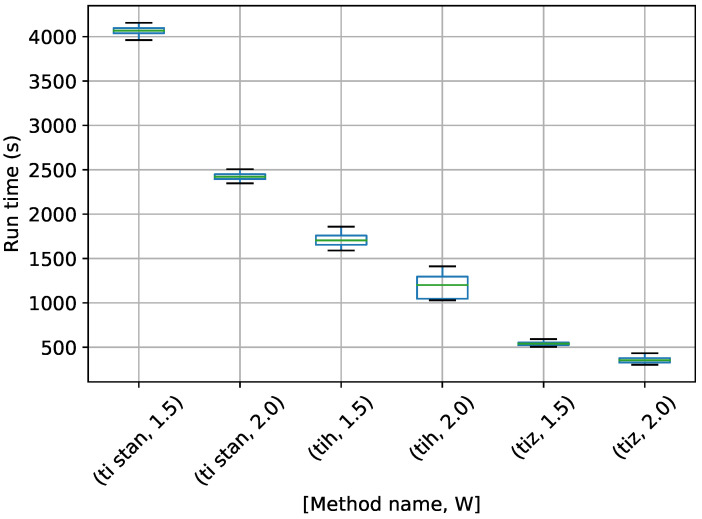
Box-plot of run time for the J=3 stationary frequency model for TI-Stan, TI-BSS-H, and TI-BSS-Z, for two values of *W*. TI-Stan with W=xx is denoted by ti stan, xx; TI-BSS-H with W=xx is denoted by tih, xx; and TI-BSS-Z with W=xx is denoted by tiz, xx.

Box-plots of the run time for models assuming one, two, and three frequencies present are shown in Figure 17, Figure 18 and Figure 19.

In Figure 17, TI-Stan has the greatest run time for both values of *W*, suggesting that its adaptive sampling process had trouble efficiently sampling distributions based on this high-error model. TI-BSS-H was much faster, and TI-BSS-Z was faster still. In Figure 18, the run times of TI-Stan and TI-BSS-H are comparable. This suggests that TI-Stan was able to more effectively sample the distribution based on the lower-error model. Figure 19 shows a similar pattern in the run times to Figure 17. The fact that this model is able to fit the noise in the data (yielding especially sharp distributions) and the fact that the distribution is increasingly multi-modal as the number of frequencies increases may explain why TI-Stan took a long time to compute a result here.

#### 6.5.4. Ideal Gas Partition Function Results

Results for the ideal gas partition function problem are presented for only the TI-Stan method. This problem requires parameter counts exceeding what TI-BSS can usefully deal with. The method parameters for this problem are different from those used in the previous three examples. For this problem, S=20 number of steps per iteration of Stan were allowed, while C=24 chains were used. A similar Google Cloud instance using 32 Intel Broadwell vCPUS and 28.8 GB of RAM was used here.

Table 5 shows the results for an ideal gas partition function problem. The first column indicates the value of *W* used by TI-Stan, the second column shows the number of dimensions, the third and fourth columns show statistics for the relative error of the numerical result with respect to the analytic value, the fifth and sixth columns show statistics for the $\log\tilde{Z}$ results, and the seventh column shows the analytic value of $\log\tilde{Z}$ for each value of *N* as given in Equation (30). Table 6 shows the run time statistics for each value of *W* and *N*.

These results demonstrate that TI-Stan can estimate thermodynamic quantities of interest with a small amount of error. They also show that the method can produce useful results with distributions with up to 1000 dimensions.

## 7. Conclusions

This article has presented three distinct model comparison algorithms based on thermodynamic integration: TI-BSS-H, TI-BSS-Z, and TI-Stan. Among these, TI-Stan is the most versatile and robust, providing log-evidence estimates in a reasonable amount of time for a wide range of example problems. TI-BSS-H is also a robust technique, yielding accurate estimates for three of the example problems; however, TI-BSS-H has trouble yielding results in a timely manner when the number of dimensions increases beyond a certain point. TI-BSS-Z is less robust, not yielding results at all in many of the examples presented. For problems with less troublesome distributions, it may be worth considering as an option, especially because its run time tends to be significantly less than TI-BSS-H.

## Figures and Tables

**Table 1:** Prior bounds for multiple stationary frequency model parameters.

	Lower Bound	Upper Bound
$A_{j}$	−2	2
$B_{j}$	−2	2
$f_{j}$	0 Hz	6.4 Hz

**Table 2:** Parameters used to generate simulated signal.

*j*	$A_{j}$	$B_{j}$	$f_{j}$ (Hz)
1	1.0	0.0	3.1
2	1.0	0.0	5.9

**Table 3:** Parameters for TI-BSS examples.

Parameter	Value	Definition
*S*	200	Number of binary slice sampling steps
*M*	2	Number of combined binary slice sampling and leapfrog steps
*C*	256	Number of chains
*B*	32	Number of bits per parameter in SFC

**Table 4:** Parameters for TI-Stan examples.

Parameter	Value	Definition
*S*	200	Number of steps allowed in Stan
*C*	256	Number of chains

**Table 5:** Ideal Gas Partition Function $\log\tilde{Z}$ results for TI-Stan for two values of *W*. Twenty TI-Stan runs were completed for each value of *W* and *N*.

*W*	*N*	Mean Relative Error	StDev Relative Error	Mean $\log\tilde{Z}$	StDev $\log\tilde{Z}$	Analytic $\log\tilde{Z}$ (30)
1.05	12	0.52%	0.37%	−12.43	0.0565	−12.49
"	102	0.51%	0.20%	−118.20	0.235	−118.81
"	1002	0.62%	0.26%	−1184.16	3.04	−1191.51
1.5	12	2.94%	1.72%	−12.12	0.215	−12.49
"	102	3.39%	1.44%	−114.78	1.71	−118.81
"	1002	4.50%	1.40%	−1137.83	16.73	−1191.51

**Table 6:** Ideal Gas Partition Function $\log\tilde{Z}$ run times for TI-Stan for two values of *W*. Twenty TI-Stan runs were completed for each value of *W* and *N*.

*W*	*N*	Mean Run Time (s)	StDev Run Time (s)
1.05	12	69.80	3.01
"	102	230.56	5.74
"	1002	2076.28	44.82
1.5	12	8.42	0.43
"	102	27.87	1.44
"	1002	247.53	6.80

## Abbreviations

NS	Nested sampling
NS-CC	Combined-chain nested sampling
NS-MR	Multiple-replacement nested sampling
TI	Thermodynamic integration
BSS	Binary slice sampling
TI-Stan	Thermodynamic integration with Stan
TI-BSS	Thermodynamic integration with binary slice sampling
TI-BSS-H	Thermodynamic integration with binary slice sampling and the Hilbert curve
TI-BSS-Z	Thermodynamic integration with binary slice sampling and the Z-order curve
HMC	Hamiltonian Monte Carlo
NUTS	No U Turn Sampler
MCMC	Markov chain Monte Carlo
CDF	cumulative distribution function
